# Supplementary material for: Exploring standardisation, monitoring and training of medical devices in assisted vaginal birth studies: protocol for a systematic review
Source: BMJ Open. 2019 Apr 14;9(4):e028300. doi: 10.1136/bmjopen-2018-028300 (PMC6500334; doi:10.1136/bmjopen-2018-028300)
Supplement: Supplementary data [file bmjopen-2018-028300supp001.pdf]

SUPPLEMENTARY FILE

Medline search strategy

Medline via HDAS

Search date: 15.06.2018

1 exp "DELIVERY, OBSTETRIC"/

2 exp "LABOR, OBSTETRIC"/

3 PARTURITION/

4 (labor OR labour OR birth OR childbirth OR delivery).ti,ab

5 (1 OR 2 OR 3 OR 4)

6 exp "EXTRACTION, OBSTETRICAL"/

7 "OBSTETRICAL FORCEPS"/

8 (forceps).ti,ab

9 (ventouse).ti,ab

10 ("suction cup").ti,ab

11 (kiwi OR malmstrom).ti,ab

12 (vacuum).ti,ab

13 (odon).ti,ab

14 ((operative OR instrumental OR assisted) OADJ1 (delivery OR birth)).ti,ab

15 (6 OR 7 OR 8 OR 9 OR 10 OR 11 OR 12 OR 13 OR 14)

16 (randomized controlled trial).pt

17 (controlled clinical trial).pt

18 (multicenter study).pt

19 (pragmatic clinical trial).pt

20 (randomis* OR randomiz* OR randomly).ti,ab

21 (trial OR multicenter OR "multi center" OR multicentre OR "multi centre").ti

22 NON-RANDOMIZED CONTROLLED TRIALS AS TOPIC/

23 "FEASIBILITY STUDIES"/

24 "PILOT PROJECTS"/

25 (pilot OR feasibility).ti,ab

26 (simulat*).ti,ab

27 exp "SIMULATION TRAINING"/

28 (16 OR 17 OR 18 OR 19 OR 20 OR 21 OR 22 OR 23 OR 24 OR 25 OR 26 OR 27)

29 (5 AND 15 AND 28)
