# Supplementary material for: Exploring standardisation, monitoring and training of medical devices in assisted vaginal birth studies: protocol for a systematic review
Source: BMJ Open. 2019 Apr 14;9(4):e028300. doi: 10.1136/bmjopen-2018-028300 (PMC6500334; doi:10.1136/bmjopen-2018-028300)
Supplement: Author's manuscript [file bmjopen-2018-028300.draft_revisions.pdf]

BMJ Open

BMJ Open is committed to open peer review. As part of this commitment we make the peer review history of every article we publish publicly available.

When an article is published we post the peer reviewers' comments and the authors' responses online. We also post the versions of the paper that were used during peer review. These are the versions that the peer review comments apply to.

The versions of the paper that follow are the versions that were submitted during the peer review process. They are not the versions of record or the final published versions. They should not be cited or distributed as the published version of this manuscript.

BMJ Open is an open access journal and the full, final, typeset and author-corrected version of record of the manuscript is available on our site with no access controls, subscription charges or pay-per-view fees (http://bmjopen.bmj.com).

If you have any questions on BMJ Open's open peer review process please email editorial.bmjopen@bmj.com

BMJ Open

Exploring standardisation, monitoring and training of medical devices in assisted vaginal birth studies: protocol for a systematic review

Journal:	*BMJ Open*
Manuscript ID	bmjopen-2018-028300
Article Type:	Protocol
Date Submitted by the Author:	30-Nov-2018
Complete List of Authors:	Hotton, Emily; University of Bristol, Translational Health Sciences; North Bristol NHS Trust, Women's and Children's Research<br>Renwick, Sophie; North Bristol NHS Trust, Women's and Children's Research<br>Barnard, Katie; North Bristol NHS Trust, Women's and Children's Research<br>Lenguerrand, Erik; University of Bristol School of Clinical Science, School of Clinical Sciences, Musculoskeletal Research Unit<br>Wade, Julia; University of Bristol, School of Social and Community Medicine<br>Draycott, Tim; North Bristol NHS Trust, Womens & Childrens Health<br>Crofts, Joanna; North Bristol NHS Trust, Women's and Children's Research<br>Blencowe, Natalie; University of Bristol, Centre for Surgical Research, School of Social and Community Medicine
Keywords:	Assisted vaginal birth, Complex interventions, Intervention standardisation, Intervention fidelity, Randomised controlled trials

SCHOLARONE™
Manuscripts

Exploring standardisation, monitoring and training of medical devices in assisted vaginal birth studies: protocol for a systematic review

Emily J Hotton [1,2 *], Sophie Renwick [2], Katie Barnard [2], Erik Lenguerrand [1,2], Julia Wade [3], Tim J Draycott [2], Joanna F Crofts [2], Natalie S Blencowe[4,5]

[1] Translational Health Sciences, University of Bristol, Bristol, UK

[2] Southmead Hospital, North Bristol NHS Trust, Bristol, UK

[3] Population Health Sciences, University of Bristol, Bristol, UK

[4] Centre for Surgical Research, Population Health Sciences, University of Bristol, Bristol, UK

[5] University Hospitals Bristol NHS Foundation Trust, Bristol, UK

*** Corresponding Author:** Dr Emily J Hotton

Women's Health

The Chilterns

Southmead Hospital

Bristol, UK

emily.hotton@nhs.net

Full word count (including abstract): 2583

V6.0 20/11/2018

For peer review only

ABSTRACT

Introduction

Assisted vaginal birth (AVB) can markedly improve maternal and neonatal outcomes arising from complications in the second stage of labour. Historically, both forceps and ventouse devices have been used to assist birth; however, they are not without risk and are associated with complications such as cephalohaematoma, retinal haemorrhage and perineal trauma. As new devices are developed to overcome the limitations of existing techniques, it is necessary to establish their efficacy and effectiveness within randomised controlled trials. A major challenge of evaluating complex interventions (i.e. invasive procedures/devices used to assist vaginal birth) is ensuring they are delivered as intended. It can be difficult to standardise intervention delivery and monitor fidelity, and account for the varying expertise of clinicians (accoucher expertise). This paper describes the protocol for a systematic review aiming to investigate the reporting of device standardisation, monitoring and training in trials evaluating complex interventions, using AVB as a case study.

Methods and analysis

Relevant keywords and subject headings will be used to conduct a comprehensive search of Medline, EMBASE, Cochrane Central Register of Controlled Trials, Cumulative Index of Nursing and Allied Health Literature and ClinicalTrials.gov, for randomised controlled trials and pilot/feasibility studies evaluating assisted vaginal birth. Abstracts will be screened and full-text articles of eligible studies reviewed for inclusion. Information relating to the following categories will be extracted: standardisation of device use (i.e. descriptions of operative steps, including mandatory/flexible parameters); monitoring of intervention delivery (i.e. intervention fidelity, confirming that an intervention is delivered as intended), and accoucher expertise (i.e. entry criteria for participation, training programmes, previous experience with the device). Risk of bias of included studies will be assessed.

For peer review only - http://bmjopen.bmj.com/site/about/guidelines.xhtml

Ethics/dissemination

Ethical approval is not required because primary data will not be collected. Findings will be disseminated by publishing in a peer-reviewed journal and presentations at relevant conferences.

Abstract word count: 331

For peer review only

ARTICLE SUMMARY – STRENGTHS AND LIMITATIONS OF THIS STUDY

- This review will improve the understanding of how complex interventions (such as use of devices to assist vaginal birth) are delivered in RCTs which will help with future trial design
- Specifically, the review will summarise reporting standards relating to standardisation and monitoring of intervention delivery, and ways in which trials describe and account for clinician expertise in RCTs involving devices
- No language limitations have been set, ensuring that the review is as comprehensive and generalisable as possible.
- This review focuses only on randomised controlled trials and pilot/feasibility studies, meaning that information from other study designs may be missed.

Keywords: assisted vaginal birth, complex interventions, intervention standardisation, intervention fidelity, randomised controlled trials.

For peer review only - http://bmjopen.bmj.com/site/about/guidelines.xhtml

INTRODUCTION

Assisted vaginal birth (AVB) is a vital procedure that, in skilled hands, can markedly reduce maternal and neonatal complications in the second stage of labour.(1) In the UK, approximately one in eight women require an AVB, which typically involves forceps and/or ventouse devices.(2) However, AVB is not without risk. A forceps assisted birth confers an increased risk of perineal and vaginal trauma(3,4) as well as faecal incontinence.(4,5) Ventouse assisted births have a failure rate of approximately 30% as well as being associated with neonatal subgaleal haematoma and intracranial haemorrhage, leading to a statutory warning in 2015 by the Food & Drugs Administration.(4) These problems, together with the threat of litigation, have contributed to a reduction in AVB rates worldwide. There has been a corresponding increase in Caesarean section rates, despite the fact that AVB often provides better outcomes at full dilation and prevents future problems such as increased risk of abnormal placentation, scar rupture and unexplained stillbirth in subsequent pregnancies.(6,7) Novel AVB devices may be able to address these known risks and attempt to transform the falling AVB rates worldwide. One example is the BD Odon Device. The device has an air cuff which, once placed around the baby's head, is inflated. To assist the birth of the baby the accoucher then applies traction on the sleeve, which is attached to the air cuff (Figure 1). In contrast to the ventouse, which operates by exerting negative pressure on the baby's head, the BD Odon Device exerts positive pressure via the air cuff. It is hypothesised that this may reduce neonatal intracranial bleeding, and that the circumferential positioning of the air cuff may reduce instrumental failure rates.

Despite the perceived benefits of novel devices such as the BD Odon Device, novel devices are susceptible to 'optimism bias'. Optimism bias refers to the unjustified belief in 'new or novel' innovations.(8) It is therefore necessary for all pioneering technologies to undergo rigorous evaluation to ensure that the benefits and harms are fully investigated and establish whether they are better than the standard devices used in clinical practice. Many expert panels, including the European Clinical Research Infrastructure Network (ECRIN), have suggested that more rigorous clinical evaluation of medical devices within randomised controlled trials (RCTs) is

For peer review only - http://bmjopen.bmj.com/site/about/guidelines.xhtml

required.(9-12) Currently, however, the pathway for evaluating novel procedures and devices is less distinct than that for pharmaceutical products, and specific barriers have been identified in undertaking RCTs in this area.(13) A major challenge is that they are considered to be complex interventions - defined as those with multiple interacting components that can act independently or interdependently to influence outcomes. This can create difficulties in establishing how the intervention should be delivered (standardisation) and ascertaining whether it is actually delivered as intended (intervention fidelity). An additional challenge is that the delivery of complex interventions can be influenced by clinicians' skill.

These issues have been acknowledged in reporting guidance documents such as the CONSORT extension for non-pharmacological treatments (CONSORT-NPT).(14) CONSORT-NPT suggests that 'precise details of the experimental treatment', 'details on whether and how the interventions were standardised', 'eligibility criteria for care providers', 'the number of care providers', 'a description of care providers expertise and qualification' and 'the number of patients treated by each care provider' are reported.(14) Additionally, 'details of whether and how adherence of care providers to the protocol and of participants to interventions was assessed' is recommended.(14) Provision of this information is recommended to improve the quality of trial design and to enable successful interventions to be replicated in practice, improving the contextualisation of findings and reducing research waste. Currently, however, it is uncertain as to whether these reporting standards are met in RCTs involving complex interventions such as devices. This study therefore aims to investigate the quality of reporting of intervention standardisation, monitoring and clinician expertise in trials involving devices, using AVB as a case study.

METHODS AND ANALYSIS

The review will be conducted in line with the Preferred Reporting Items for Systematic Reviews and Meta-Analysis (PRISMA) checklist.(15)

For peer review only - http://bmjopen.bmj.com/site/about/guidelines.xhtml

Eligibility criteria

Feasibility studies, pilot studies and RCTs will be included in the review if they meet the following inclusion criteria:

Participants

All females of any age having an AVB. Studies involving simulated patients or animals will also be included.

Intervention

AVB by forceps, vacuum extraction or a novel assisted birth device. All devices will be considered and will not be limited to a single type or manufacturer.

Comparator(s)

Comparator groups will include spontaneous vaginal birth, AVB using any device, or Caesarean section. Pilot/feasibility studies without a comparator group will also be included.

Outcome(s)

Reporting standards relating to standardisation of device use, monitoring of whether the device was used as intended (intervention fidelity), and details of accoucher expertise will be extracted. Information about the 'success' and 'failure' rates of the device, and adverse events, will also be collected.

Search strategy and study selection

We will systematically search for RCTs involving AVB device(s) in Medline, EMBASE, Cochrane Central Register of Controlled Trials (CENTRAL), Cumulative Index of Nursing and Allied Health Literature (CINAHL) and ClinicalTrials.gov databases from inception to November 2018. The computer-based searches will combine free text and subject headings (see Supplementary File).

For peer review only

For peer review only - http://bmjopen.bmj.com/site/about/guidelines.xhtml

Reference lists of included studies will be searched for additional relevant articles, including published protocols. There will be no restrictions on language.

Identification and selection of papers

A customised inclusion/exclusion form will be used to screen abstracts and provide an audit trail. Titles and abstracts will be screened independently by two authors (EH and NB). Any conflicts will be resolved by discussion.

The full-text versions of papers retained after title and abstract screening will be screened for further assessment of their eligibility for inclusion.

Data extraction and management

Data will be extracted independently by at least two assessors for each paper (EH, SR and NB). A customised data extraction form will be used to collect relevant data from each paper. Data of interest will include general study details (author, year of publication, country of origin of study), details of study design (RCT, pilot or feasibility study), the number of participating centres and the total number of participants.

Standardisation of intervention delivery

Details of the device(s) and comparator(s) will be extracted. These will include verbatim descriptions relating to how the device should be delivered (including technical or operative steps) and how/whether this was standardised within the study. Details concerning the criteria for using the device, such as any mandatory, prohibited or flexible parameters, will be documented in accordance with an existing typology for considering standardisation of interventional procedures.(16) Finally, assessors will record judgements about whether enough information is provided to be able to replicate device use in routine practice (yes/no/unsure).

Monitoring of whether the device was used as intended (intervention fidelity)

For peer review only - http://bmjopen.bmj.com/site/about/guidelines.xhtml
For peer review only

Any reporting of whether the device was used as intended (intervention fidelity), will be reported. Details of how intervention fidelity was measured will be documented (for example, within case report forms).

Accoucher expertise

The number of accouchers participating in the study, and delivering interventions in each trial group, will be recorded. If provided, the total number of births (and AVBs) in each study centre will be reported. Reporting of any information about accoucher expertise will be recorded including their grade, previous experience with the device(s) under investigation, and any protocols for supervision when using the device. Attempts to account for a potential learning curve in device delivery (for example, trial entry criteria for accouchers such as a pre-specified number of deliveries) will be recorded, together with information about accoucher training (e.g. mandatory courses, videos or other materials). Finally, accoucher related outcomes such as competence, confidence or knowledge will be extracted.

Device success, failure and safety

Details of whether the device was used successfully will be recorded, together with information about 'harms' or 'adverse events' in either women or their babies. Information about causes or reasons for these events will be extracted verbatim.

Assessment of study quality

The Cochrane Risk of Bias tool will be used to evaluate bias in RCTs, and pilot or feasibility studies that involved randomisation.(17) Non-randomised pilot and feasibility studies will be assessed by evaluating bias related to the process of trial recruitment, documentation of protocol non-adherence, reporting of a primary outcome, description of clear objectives and description of clear progression criteria.

Data synthesis and statistical analysis

For peer review only - http://bmjopen.bmj.com/site/about/guidelines.xhtml

Data will be entered into a custom database. A narrative synthesis will summarise the findings. Any further data synthesis (such as meta-analyses) will depend on the number and quality of studies identified.

ETHICS AND DISSEMINATION

The completed systematic review will be published in a peer-reviewed journal and presented at appropriate conferences. This protocol can further be adapted for the analysis of other devices within obstetrics and surgery.

This systematic review will provide important information surrounding the quality of reporting in RCTs evaluating devices for AVB, relating to how device use is standardised in trials (standardisation), whether devices are used in trials as intended (monitoring/intervention fidelity) and what the level of accoucher training is. The findings will inform the design of future pilot/feasibility studies and/or RCTs in this area, by optimising the way that device use is standardised and monitored, and accoucher expertise is accounted for.

For peer review only

For peer review only - http://bmjopen.bmj.com/site/about/guidelines.xhtml

REFERENCES

1. Hirshberg A, Srinivas SK. Role of operative vaginal deliveries in prevention of cesarean deliveries. Clin Obstet Gynecol. 2015;58(2):256–62.

2. RCOG. Information for you. An assisted vaginal birth. 2014.

3. O'Mahony F, Hofmeyr GJ, Menon V. Choice of instruments for assisted vaginal delivery. O'Mahony F, editor. Cochrane Database of Systematic Reviews. Chichester, UK: John Wiley & Sons, Ltd; 2010;16(11):201–107.

4. RCOG. Operative Vaginal Delivery: Green–top Guideline No. 26. London: RCOG; 2011.

5. Patel RR, Murphy DJ. Forceps delivery in modern obstetric practice. BMJ. British Medical Journal Publishing Group; 2004;328(7451):1302–5.

6. Clark EAS, Silver RM. Long-term maternal morbidity associated with repeat cesarean delivery. Am J Obstet Gynecol. 2011 Dec;205(6 Suppl):S2–10.

7. Moraitis AA, Oliver-Williams C, Wood AM, Fleming M, Pell JP, Smith G. Previous caesarean delivery and the risk of unexplained stillbirth: retrospective cohort study and meta-analysis. BJOG: An International Journal of Obstetrics & Gynaecology. 2015;122(11):1467–74.

8. Chalmers I, Matthews R. What are the implications of optimism bias in clinical research? Lancet. 2006;367(9509):449–50.

9. Neugebauer EAM, Rath A, Antoine S-L, Eikermann M, Seidel D, Koenen C, et al. Specific barriers to the conduct of randomised clinical trials on medical devices. Trials. 2017;18(1):427.

10. Heneghan C, Thompson M, Billingsley M, Cohen D. Medical-device recalls in the UK and the device-regulation process: retrospective review of safety notices and alerts. BMJ Open. British Medical Journal Publishing Group; 2011;1(1):e000155–5.

11. Cohen D, Billingsley M. Europeans are left to their own devices. BMJ. 2011;342:d2748.

12. Eikermann M, Gluud C, Perleth M, Wild C, Sauerland S, Gutierrez-Ibarluzea I, et al. Commentary: Europe needs a central, transparent, and evidence based regulation process for devices. BMJ. 2013;346:f2771.

13. Blencowe NS, Cook JA, Pinkney T, Rogers C, Reeves BC, Blazeby JM. Delivering successful randomized controlled trials in surgery: Methods to optimize collaboration and study design. Clin Trials. 2017;14(2):211–

For peer review only - http://bmjopen.bmj.com/site/about/guidelines.xhtml

8.

14. Boutron I, Altman DG, Moher D, Schulz KF, Ravaud P, CONSORT NPT Group. CONSORT Statement for Randomized Trials of Nonpharmacologic Treatments: A 2017 Update and a CONSORT Extension for Nonpharmacologic Trial Abstracts. Ann Intern Med. American College of Physicians; 2017;167(1):40–7.

15. Moher D, Liberati A, Tetzlaff J, Altman DG, Grp P. Preferred reporting items for systematic reviews and meta-analyses: the PRISMA statement. BMJ. British Medical Journal Publishing Group; 2009;339(jul21 1):–b2535.

16. Blencowe NS, Boddy AP, Harris A, Hanna T, Whiting P, Cook JA, et al. Systematic review of intervention design and delivery in pragmatic and explanatory surgical randomized clinical trials. Br J Surg. 2015 Jun 3;102(9):1037–47.

17. Higgins JPT, Altman DG, Gøtzsche PC, Jüni P, Moher D, Oxman AD, et al. The Cochrane Collaboration's tool for assessing risk of bias in randomised trials. BMJ. 2011;343:d5928.

AUTHORS' CONTRIBUTIONS

EH and NB initiated and designed the study with input from all other authors. EH and NB drafted the manuscript. All authors contributed to revisions of the manuscript and approved the final version.

FUNDING STATEMENT

This work was supported by the Bill & Melinda Gates Foundation [grant number OPP1184825].

EH, SR, JFC, TD are employees of North Bristol NHS Trust, which receives funding from PROMPT Maternity Foundation (PMF) to pay part of their salaries. PMF has received funds from BD, manufacturer of the Odon Device. EL is an employee of the University of Bristol, which receives funding from PROMPT Maternity Foundation (PMF) to pay part of EL's salary. NB is an NIHR Clinical Lecturer. This study is being supported by the NIHR Biomedical Research Centre at the University Hospitals Bristol NHS Foundation Trust and the University of Bristol, and the MRC ConDuCT-II

For peer review only - http://bmjopen.bmj.com/site/about/guidelines.xhtml

(Collaboration and innovation for Difficult and Complex randomised controlled Trials In Invasive procedures) Hub for Trials Methodology Research (MR/K025643/1).

This work was supported by the Bill & Melinda Gates Foundation [grant number OPP1184825].

COMPETING INTERESTS STATEMENT

None declared.

For peer review only

SUPPLEMENTARY FILE

Medline search strategy

Medline via HDAS
Search date: 15.06.2018

1	exp "DELIVERY, OBSTETRIC"/
2	exp "LABOR, OBSTETRIC"/
3	PARTURITION/
4	(labor OR labour OR birth OR childbirth OR delivery).ti,ab
5	(1 OR 2 OR 3 OR 4)
6	exp "EXTRACTION, OBSTETRICAL"/
7	"OBSTETRICAL FORCEPS"/
8	(forceps).ti,ab
9	(ventouse).ti,ab
10	("suction cup").ti,ab
11	(kiwi OR malmstrom).ti,ab
12	(vacuum).ti,ab
13	(odon).ti,ab
14	((operative OR instrumental OR assisted) OADJ1 (delivery OR birth)).ti,ab
15	(6 OR 7 OR 8 OR 9 OR 10 OR 11 OR 12 OR 13 OR 14)
16	(randomized controlled trial).pt
17	(controlled clinical trial).pt
18	(multicenter study).pt
19	(pragmatic clinical trial).pt
20	(randomis* OR randomiz* OR randomly).ti,ab
21	(trial OR multicenter OR "multi center" OR multicentre OR "multi centre").ti
22	NON-RANDOMIZED CONTROLLED TRIALS AS TOPIC/
23	"FEASIBILITY STUDIES"/
24	"PILOT PROJECTS"/

For peer review only - http://bmjopen.bmj.com/site/about/guidelines.xhtml

25	(pilot OR feasibility).ti,ab
26	(simulat*).ti,ab
27	exp "SIMULATION TRAINING"/
28	(16 OR 17 OR 18 OR 19 OR 20 OR 21 OR 22 OR 23 OR 24 OR 25 OR 26 OR 27)
29	(5 AND 15 AND 28)

Figure 1. BD Odon Device components

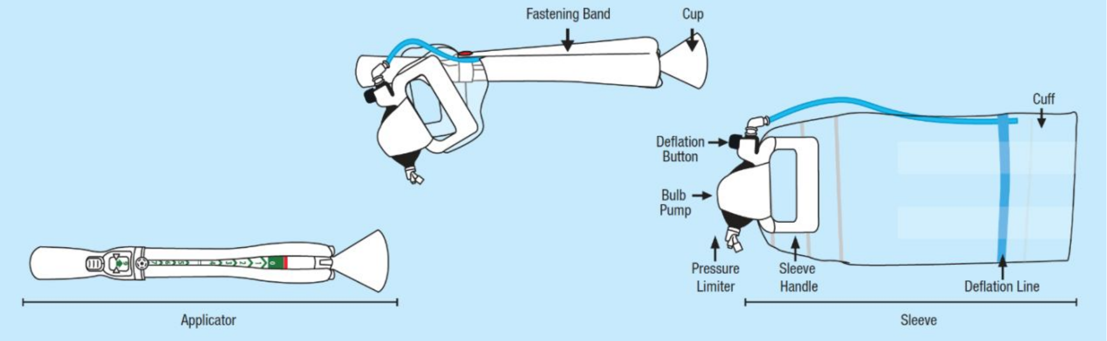

For peer review only - http://bmjopen.bmj.com/site/about/guidelines.xhtml

Exploring standardisation, monitoring and training of medical devices in assisted vaginal birth studies: protocol for a systematic review

Journal:	*BMJ Open*
Manuscript ID	bmjopen-2018-028300.R1
Article Type:	Protocol
Date Submitted by the Author:	04-Mar-2019
Complete List of Authors:	Hotton, Emily; University of Bristol, Translational Health Sciences; North Bristol NHS Trust, Women's and Children's Research<br>Renwick, Sophie; North Bristol NHS Trust, Women's and Children's Research<br>Barnard, Katie; North Bristol NHS Trust, Women's and Children's Research<br>Lenguerrand, Erik; University of Bristol School of Clinical Science, School of Clinical Sciences, Musculoskeletal Research Unit<br>Wade, Julia; University of Bristol, School of Social and Community Medicine<br>Draycott, Tim; North Bristol NHS Trust, Womens & Childrens Health<br>Crofts, Joanna; North Bristol NHS Trust, Women's and Children's Research<br>Blencowe, Natalie; University of Bristol, Centre for Surgical Research, School of Social and Community Medicine
**Primary Subject Heading**:	Obstetrics and gynaecology
Secondary Subject Heading:	Evidence based practice
Keywords:	Assisted vaginal birth, Complex interventions, Intervention standardisation, Intervention fidelity, Randomised controlled trials

SCHOLARONE™
Manuscripts

Exploring standardisation, monitoring and training of medical devices in assisted vaginal birth studies: protocol for a systematic review

Emily J Hotton [1,2 *], Sophie Renwick [2], Katie Barnard [2], Erik Lenguerrand [1,2], Julia Wade [3], Tim J Draycott [2], Joanna F Crofts [2], Natalie S Blencowe[4,5]

[1] Translational Health Sciences, University of Bristol, Bristol, UK

[2] Southmead Hospital, North Bristol NHS Trust, Bristol, UK

[3] Population Health Sciences, University of Bristol, Bristol, UK

[4] Centre for Surgical Research, Population Health Sciences, University of Bristol, Bristol, UK

[5] University Hospitals Bristol NHS Foundation Trust, Bristol, UK

*** Corresponding Author:** Dr Emily J Hotton

Women's Health

The Chilterns

Southmead Hospital

Bristol, UK

emily.hotton@nhs.net

Full word count (including abstract): 1975

03/03/2019

For peer review only

ABSTRACT

Introduction

Assisted vaginal birth (AVB) can markedly improve maternal and neonatal outcomes arising from complications in the second stage of labour. Historically, both forceps and ventouse devices have been used to assist birth; however, they are not without risk and are associated with complications such as cephalohaematoma, retinal haemorrhage and perineal trauma. As new devices are developed to overcome the limitations of existing techniques, it is necessary to establish their efficacy and effectiveness within randomised controlled trials. A major challenge of evaluating complex interventions (i.e. invasive procedures/devices used to assist vaginal birth) is ensuring they are delivered as intended. It can be difficult to standardise intervention delivery and monitor fidelity, and account for the varying expertise of clinicians (accoucher expertise). This paper describes the protocol for a systematic review aiming to investigate the reporting of device standardisation, monitoring and training in trials evaluating complex interventions, using AVB as a case study.

Methods and analysis

Relevant keywords and subject headings will be used to conduct a comprehensive search of Medline, EMBASE, Cochrane Central Register of Controlled Trials, Cumulative Index of Nursing and Allied Health Literature and ClinicalTrials.gov, for randomised controlled trials and pilot/feasibility studies evaluating assisted vaginal birth. Abstracts will be screened and full-text articles of eligible studies reviewed for inclusion. Information relating to the following categories will be extracted: standardisation of device use (i.e. descriptions of operative steps, including mandatory/flexible parameters); monitoring of intervention delivery (i.e. intervention fidelity, confirming that an intervention is delivered as intended), and accoucher expertise (i.e. entry criteria for participation, training programmes, previous experience with the device). Risk of bias of included studies will be assessed.

For peer review only - http://bmjopen.bmj.com/site/about/guidelines.xhtml

Ethics/dissemination

Ethical approval is not required because primary data will not be collected. Findings will be disseminated by publishing in a peer-reviewed journal and presentations at relevant conferences.

Abstract word count: 299

For peer review only

ARTICLE SUMMARY – STRENGTHS AND LIMITATIONS OF THIS STUDY

- This review will include all RCTs/feasibility studies evaluating assisted vaginal births, regardless of the nature of the comparator, ensuring that all assisted vaginal birth data are captured.
- Specifically, the review will summarise reporting standards relating to standardisation and monitoring of intervention delivery, and ways in which trials describe and account for clinician expertise in RCTs involving devices.
- The review is not limited to human studies, ensuring that any relevant assisted vaginal birth study is included.
- No language limitations have been set, ensuring that the review is as comprehensive and generalisable as possible.
- This review focuses only on randomised controlled trials and pilot/feasibility studies, meaning that information from other study designs may be missed.

Keywords: assisted vaginal birth, complex interventions, intervention standardisation, intervention fidelity, randomised controlled trials.

03/03/2019

INTRODUCTION

Assisted vaginal birth (AVB) is a vital procedure that, in skilled hands, can markedly reduce maternal and neonatal complications in the second stage of labour.(1) In the UK, approximately one in eight women require an AVB, which typically involves forceps and/or ventouse devices.(2) However, AVB is not without risk. A forceps assisted birth confers an increased risk of perineal and vaginal trauma(3,4) as well as faecal incontinence.(4,5) Ventouse assisted births have a failure rate of approximately 30% as well as being associated with neonatal subgaleal haematoma and intracranial haemorrhage, leading to a statutory warning in 2015 by the Food & Drugs Administration.(4) These problems, together with the threat of litigation, have contributed to a reduction in AVB rates worldwide. There has been a corresponding increase in Caesarean section rates, despite the fact that AVB often provides better outcomes at full dilation and prevents future problems such as increased risk of abnormal placentation, scar rupture and unexplained stillbirth in subsequent pregnancies.(6,7) Novel AVB devices may be able to address these known risks and attempt to transform the falling AVB rates worldwide. One example is the BD Odon Device. The device has an air cuff which, once placed around the baby's head, is inflated. To assist the birth of the baby the accoucher then applies traction on the sleeve, which is attached to the air cuff (Figure 1). In contrast to the ventouse, which operates by exerting negative pressure on the baby's head, the BD Odon Device exerts positive pressure via the air cuff. It is hypothesised that this may reduce neonatal intracranial bleeding, and that the circumferential positioning of the air cuff may reduce instrumental failure rates.

Despite the perceived benefits of novel devices such as the BD Odon Device, novel devices are susceptible to 'optimism bias'. Optimism bias refers to the unjustified belief in 'new or novel' innovations.(8) It is therefore necessary for all pioneering technologies to undergo rigorous evaluation to ensure that the benefits and harms are fully investigated and establish whether they are better than the standard devices used in clinical practice. Many expert panels, including the European Clinical Research Infrastructure Network (ECRIN), have suggested that more rigorous clinical evaluation of medical devices within randomised controlled trials (RCTs) is

For peer review only - http://bmjopen.bmj.com/site/about/guidelines.xhtml

required.(9-12) Currently, however, the pathway for evaluating novel procedures and devices is less distinct than that for pharmaceutical products, and specific barriers have been identified in undertaking RCTs in this area.(13) A major challenge is that they are considered to be complex interventions - defined as those with multiple interacting components that can act independently or interdependently to influence outcomes. This can create difficulties in establishing how the intervention should be delivered (standardisation) and ascertaining whether it is actually delivered as intended (intervention fidelity). An additional challenge is that the delivery of complex interventions can be influenced by clinicians' skill.

These issues have been acknowledged in reporting guidance documents such as the CONSORT extension for non-pharmacological treatments (CONSORT-NPT).(14) CONSORT-NPT suggests that 'precise details of the experimental treatment', 'details on whether and how the interventions were standardised', 'eligibility criteria for care providers', 'the number of care providers', 'a description of care providers expertise and qualification' and 'the number of patients treated by each care provider' are reported.(14) Additionally, 'details of whether and how adherence of care providers to the protocol and of participants to interventions was assessed' is recommended.(14) Provision of this information is recommended to improve the quality of trial design and to enable successful interventions to be replicated in practice, improving the contextualisation of findings and reducing research waste. Currently, however, it is uncertain as to whether these reporting standards are met in RCTs involving complex interventions such as devices. This study therefore aims to investigate the quality of reporting of intervention standardisation, monitoring and clinician expertise in trials involving devices, using AVB as a case study.

METHODS AND ANALYSIS

The review will be conducted in line with the Preferred Reporting Items for Systematic Reviews and Meta-Analysis (PRISMA) checklist.(15)

For peer review only - http://bmjopen.bmj.com/site/about/guidelines.xhtml

Eligibility criteria

Feasibility studies, pilot studies and RCTs will be included in the review if they meet the following inclusion criteria:

Participants

All females of any age having an AVB. Studies involving simulated patients or animals will also be included.

Intervention

AVB by forceps, vacuum extraction or a novel assisted birth device. All devices will be considered and will not be limited to a single type or manufacturer.

Comparator(s)

Comparator groups will include spontaneous vaginal birth, AVB using any device, or Caesarean section. Pilot/feasibility studies without a comparator group will also be included.

Outcome(s)

Reporting standards relating to standardisation of device use, monitoring of whether the device was used as intended (intervention fidelity), and details of accoucher expertise will be extracted. Information about the 'success' and 'failure' rates of the device, and adverse events, will also be collected.

Search strategy and study selection

We will systematically search for RCTs involving AVB device(s) in Medline, EMBASE, Cochrane Central Register of Controlled Trials (CENTRAL), Cumulative Index of Nursing and Allied Health Literature (CINAHL) and ClinicalTrials.gov databases from inception to 30th November 2018. The computer-based searches will combine free text and subject headings (see Supplementary File).

For peer review only

For peer review only - http://bmjopen.bmj.com/site/about/guidelines.xhtml

Reference lists of included studies will be searched for additional relevant articles, including published protocols. There will be no restrictions on language.

Identification and selection of papers

A customised inclusion/exclusion form will be used to screen abstracts and provide an audit trail. Titles and abstracts will be screened independently by two authors (EH and NB). Any conflicts will be resolved by discussion.

The full-text versions of papers retained after title and abstract screening will be screened for further assessment of their eligibility for inclusion.

Data extraction and management

Data will be extracted independently by at least two assessors for each paper (EH, SR and NB). A customised data extraction form will be used to collect relevant data from each paper. Data of interest will include general study details (author, year of publication, country of origin of study), details of study design (RCT, pilot or feasibility study), the number of participating centres and the total number of participants.

Standardisation of intervention delivery

Details of the device(s) and comparator(s) will be extracted. These will include verbatim descriptions relating to how the device should be delivered (including technical or operative steps) and how/whether this was standardised within the study. Details concerning the criteria for using the device, such as any mandatory, prohibited or flexible parameters, will be documented in accordance with an existing typology for considering standardisation of interventional procedures.(16) Finally, assessors will record judgements about whether enough information is provided to be able to replicate device use in routine practice (yes/no/unsure).

Monitoring of whether the device was used as intended (intervention fidelity)

For peer review only - http://bmjopen.bmj.com/site/about/guidelines.xhtml
For peer review only

Any reporting of whether the device was used as intended (intervention fidelity), will be reported. Details of how intervention fidelity was measured will be documented (for example, within case report forms).

Accoucher expertise

The number of accouchers participating in the study, and delivering interventions in each trial group, will be recorded. If provided, the total number of births (and AVBs) in each study centre will be reported. Reporting of any information about accoucher expertise will be recorded including their grade, previous experience with the device(s) under investigation, and any protocols for supervision when using the device. Attempts to account for a potential learning curve in device delivery (for example, trial entry criteria for accouchers such as a pre-specified number of deliveries) will be recorded, together with information about accoucher training (e.g. mandatory courses, videos or other materials). Finally, accoucher related outcomes such as competence, confidence or knowledge will be extracted.

Device success, failure and safety

Details of whether the device was used successfully will be recorded, together with information about 'harms' or 'adverse events' in either women or their babies. Information about causes or reasons for these events will be extracted verbatim.

Assessment of study quality

The Cochrane Risk of Bias tool will be used to evaluate bias in RCTs, and pilot or feasibility studies that involved randomisation.(17) Non-randomised pilot and feasibility studies will be assessed by evaluating bias related to the process of trial recruitment, documentation of protocol non-adherence, reporting of a primary outcome, description of clear objectives and description of clear progression criteria.

Data synthesis and statistical analysis

For peer review only - http://bmjopen.bmj.com/site/about/guidelines.xhtml

Data will be entered into a custom database. A narrative synthesis will summarise the findings. Any further data synthesis (such as meta-analyses) will depend on the number and quality of studies identified.

Patient and Public Involvement

Patients and public were not involved in the design and development of this protocol.

ETHICS AND DISSEMINATION

The completed systematic review will be published in a peer-reviewed journal and presented at appropriate conferences. This protocol can further be adapted for the analysis of other devices within obstetrics and surgery.

This systematic review will provide important information surrounding the quality of reporting in RCTs evaluating devices for AVB, relating to how device use is standardised in trials (standardisation), whether devices are used in trials as intended (monitoring/intervention fidelity) and what the level of accoucher training is. The findings will inform the design of future pilot/feasibility studies and/or RCTs in this area, by optimising the way that device use is standardised and monitored, and accoucher expertise is accounted for.

Figure 1. BD Odon Device components

For peer review only - http://bmjopen.bmj.com/site/about/guidelines.xhtml
For peer review only

REFERENCES

1. Hirshberg A, Srinivas SK. Role of operative vaginal deliveries in prevention of cesarean deliveries. Clin Obstet Gynecol. 2015;58(2):256–62.

2. RCOG. Information for you. An assisted vaginal birth. 2014.

3. O'Mahony F, Hofmeyr GJ, Menon V. Choice of instruments for assisted vaginal delivery. O'Mahony F, editor. Cochrane Database of Systematic Reviews. Chichester, UK: John Wiley & Sons, Ltd; 2010;16(11):201–107.

4. RCOG. Operative Vaginal Delivery: Green–top Guideline No. 26. London: RCOG; 2011.

5. Patel RR, Murphy DJ. Forceps delivery in modern obstetric practice. BMJ. British Medical Journal Publishing Group; 2004;328(7451):1302–5.

6. Clark EAS, Silver RM. Long-term maternal morbidity associated with repeat cesarean delivery. Am J Obstet Gynecol. 2011 Dec;205(6 Suppl):S2–10.

7. Moraitis AA, Oliver-Williams C, Wood AM, Fleming M, Pell JP, Smith G. Previous caesarean delivery and the risk of unexplained stillbirth: retrospective cohort study and meta-analysis. BJOG: An International Journal of Obstetrics & Gynaecology. 2015;122(11):1467–74.

8. Chalmers I, Matthews R. What are the implications of optimism bias in clinical research? Lancet. 2006;367(9509):449–50.

9. Neugebauer EAM, Rath A, Antoine S-L, Eikermann M, Seidel D, Koenen C, et al. Specific barriers to the conduct of randomised clinical trials on medical devices. Trials. 2017;18(1):427.

10. Heneghan C, Thompson M, Billingsley M, Cohen D. Medical-device recalls in the UK and the device-regulation process: retrospective review of safety notices and alerts. BMJ Open. British Medical Journal Publishing Group; 2011;1(1):e000155–5.

11. Cohen D, Billingsley M. Europeans are left to their own devices. BMJ. 2011;342:d2748.

12. Eikermann M, Gluud C, Perleth M, Wild C, Sauerland S, Gutierrez-Ibarluzea I, et al. Commentary: Europe needs a central, transparent, and evidence based regulation process for devices. BMJ. 2013;346:f2771.

13. Blencowe NS, Cook JA, Pinkney T, Rogers C, Reeves BC, Blazeby JM. Delivering successful randomized controlled trials in surgery: Methods to optimize collaboration and study design. Clin Trials. 2017;14(2):211–

For peer review only - http://bmjopen.bmj.com/site/about/guidelines.xhtml

8.

14. Boutron I, Altman DG, Moher D, Schulz KF, Ravaud P, CONSORT NPT Group. CONSORT Statement for Randomized Trials of Nonpharmacologic Treatments: A 2017 Update and a CONSORT Extension for Nonpharmacologic Trial Abstracts. Ann Intern Med. American College of Physicians; 2017;167(1):40–7.

15. Moher D, Liberati A, Tetzlaff J, Altman DG, Grp P. Preferred reporting items for systematic reviews and meta-analyses: the PRISMA statement. BMJ. British Medical Journal Publishing Group; 2009;339(jul21 1):–b2535.

16. Blencowe NS, Boddy AP, Harris A, Hanna T, Whiting P, Cook JA, et al. Systematic review of intervention design and delivery in pragmatic and explanatory surgical randomized clinical trials. Br J Surg. 2015 Jun 3;102(9):1037–47.

17. Higgins JPT, Altman DG, Gøtzsche PC, Jüni P, Moher D, Oxman AD, et al. The Cochrane Collaboration's tool for assessing risk of bias in randomised trials. BMJ. 2011;343:d5928.

AUTHORS' CONTRIBUTIONS

EH and NB initiated and designed the study with methodology input from EL, JW, TD and JC. EH and SR performed the data collection. KB performed the database searches. EH and NB drafted the manuscript with input from SR, KB, EL, JW and JC. All authors contributed to revisions of the manuscript and approved the final version.

FUNDING STATEMENT

This work was supported by the Bill & Melinda Gates Foundation [grant number OPP1184825].

EH, SR, JFC, TD are employees of North Bristol NHS Trust, which receives funding from PROMPT Maternity Foundation (PMF) to pay part of their salaries. PMF has received funds from BD, manufacturer of the Odon Device. EL is an employee of the University of Bristol, which receives funding from PROMPT Maternity Foundation (PMF) to pay part of EL's salary. NB is an NIHR Clinical Lecturer. This study is being

For peer review only - http://bmjopen.bmj.com/site/about/guidelines.xhtml

For peer review only

supported by the NIHR Biomedical Research Centre at the University Hospitals Bristol NHS Foundation Trust and the University of Bristol, and the MRC ConDuCT-II (Collaboration and innovation for Difficult and Complex randomised controlled Trials In Invasive procedures) Hub for Trials Methodology Research (MR/K025643/1).

COMPETING INTERESTS STATEMENT

None declared.

For peer review only

For peer review only - http://bmjopen.bmj.com/site/about/guidelines.xhtml

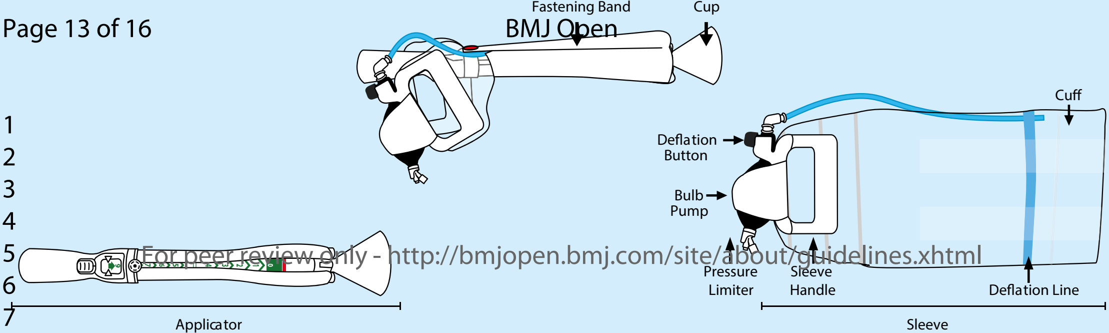

For peer review only - http://bmjopen.bmj.com/site/about/guidelines.xhtml

SUPPLEMENTARY FILE

Medline search strategy

Medline via HDAS

Search date: 15.06.2018

1 exp "DELIVERY, OBSTETRIC"/
2 exp "LABOR, OBSTETRIC"/
3 PARTURITION/
4 (labor OR labour OR birth OR childbirth OR delivery).ti,ab
5 (1 OR 2 OR 3 OR 4)
6 exp "EXTRACTION, OBSTETRICAL"/
7 "OBSTETRICAL FORCEPS"/
8 (forceps).ti,ab
9 (ventouse).ti,ab
10 ("suction cup").ti,ab
11 (kiwi OR malmstrom).ti,ab
12 (vacuum).ti,ab
13 (odon).ti,ab
14 ((operative OR instrumental OR assisted) OADJ1 (delivery OR birth)).ti,ab
15 (6 OR 7 OR 8 OR 9 OR 10 OR 11 OR 12 OR 13 OR 14)
16 (randomized controlled trial).pt
17 (controlled clinical trial).pt
18 (multicenter study).pt
19 (pragmatic clinical trial).pt
20 (randomis* OR randomiz* OR randomly).ti,ab
21 (trial OR multicenter OR "multi center" OR multicentre OR "multi centre").ti
22 NON-RANDOMIZED CONTROLLED TRIALS AS TOPIC/
23 "FEASIBILITY STUDIES"/
24 "PILOT PROJECTS"/
25 (pilot OR feasibility).ti,ab
26 (simulat*).ti,ab
27 exp "SIMULATION TRAINING"/
28 (16 OR 17 OR 18 OR 19 OR 20 OR 21 OR 22 OR 23 OR 24 OR 25 OR 26 OR 27)
29 (5 AND 15 AND 28)

For peer review only

PRISMA-P (Preferred Reporting Items for Systematic review and Meta-Analysis Protocols) 2015 checklist: recommended items to address in a systematic review protocol*

Section and topic	Item No	Checklist item		
**ADMINISTRATIVE INFORMATION**			**Page**	**Line**
Title:				
Identification	1a	Identify the report as a protocol of a systematic review	1	3
Update	1b	If the protocol is for an update of a previous systematic review, identify as such	N/A	
Registration	2	If registered, provide the name of the registry (such as PROSPERO) and registration number	N/A	
Authors:				
Contact	3a	Provide name, institutional affiliation, e-mail address of all protocol authors; provide physical mailing address of corresponding author	1	5-21
Contributions	3b	Describe contributions of protocol authors and identify the guarantor of the review	11	328-331
Amendments	4	If the protocol represents an amendment of a previously completed or published protocol, identify as such and list changes; otherwise, state plan for documenting important protocol amendments	Tracked changes	
Support:				
Sources	5a	Indicate sources of financial or other support for the review	11	334-335
Sponsor	5b	Provide name for the review funder and/or sponsor	11	334-335
Role of sponsor or funder	5c	Describe roles of funder(s), sponsor(s), and/or institution(s), if any, in developing the protocol	11	337-345
**INTRODUCTION**				
Rationale	6	Describe the rationale for the review in the context of what is already known	4	146
Objectives	7	Provide an explicit statement of the question(s) the review will address with reference to participants, interventions, comparators, and outcomes (PICO)	5	160-177
**METHODS**				
Eligibility criteria	8	Specify the study characteristics (such as PICO, study design, setting, time frame) and report characteristics (such as years considered, language, publication status) to be used as criteria for eligibility for the review	5	156
Information sources	9	Describe all intended information sources (such as electronic databases, contact with study authors, trial registers or other grey literature sources) with planned dates of coverage	5	179

For peer review only

Search strategy	10	Present draft of search strategy to be used for at least one electronic database, including planned limits, such that it could be repeated	Supplementary file	
Study records:				
Data management	11a	Describe the mechanism(s) that will be used to manage records and data throughout the review	7	188
Selection process	11b	State the process that will be used for selecting studies (such as two independent reviewers) through each phase of the review (that is, screening, eligibility and inclusion in meta-analysis)	7	188
Data collection process	11c	Describe planned method of extracting data from reports (such as piloting forms, done independently, in duplicate), any processes for obtaining and confirming data from investigators	7	188
Data items	12	List and define all variables for which data will be sought (such as PICO items, funding sources), any pre-planned data assumptions and simplifications	7-8	196-234
Outcomes and prioritization	13	List and define all outcomes for which data will be sought, including prioritization of main and additional outcomes, with rationale	7-8	196-234
Risk of bias in individual studies	14	Describe anticipated methods for assessing risk of bias of individual studies, including whether this will be done at the outcome or study level, or both; state how this information will be used in data synthesis	8	236
Data synthesis	15a	Describe criteria under which study data will be quantitatively synthesised	9	245
	15b	If data are appropriate for quantitative synthesis, describe planned summary measures, methods of handling data and methods of combining data from studies, including any planned exploration of consistency (such as I^2, Kendall's τ)	N/A	
	15c	Describe any proposed additional analyses (such as sensitivity or subgroup analyses, meta-regression)	N/A	
	15d	If quantitative synthesis is not appropriate, describe the type of summary planned	9	244-246
Meta-bias(es)	16	Specify any planned assessment of meta-bias(es) (such as publication bias across studies, selective reporting within studies)	N/A	
Confidence in cumulative evidence	17	Describe how the strength of the body of evidence will be assessed (such as GRADE)	N/A	

*** It is strongly recommended that this checklist be read in conjunction with the PRISMA-P Explanation and Elaboration (cite when available) for important clarification on the items. Amendments to a review protocol should be tracked and dated.** **The copyright for PRISMA-P (including checklist) is held by the PRISMA-P Group and is distributed under a Creative Commons Attribution Licence 4.0.**

From: Shamseer L, Moher D, Clarke M, Ghersi D, Liberati A, Petticrew M, Shekelle P, Stewart L, PRISMA-P Group. Preferred reporting items for systematic review and meta-analysis protocols (PRISMA-P) 2015: elaboration and explanation. BMJ. 2015 Jan 2;349(jan02 1):g7647.

For peer review only
